# BACT: nonparametric Bayesian cell typing for single-cell spatial transcriptomics data

**DOI:** 10.1093/bib/bbae689

**Published:** 2025-01-03

**Authors:** Yinqiao Yan, Xiangyu Luo

**Affiliations:** School of Mathematics, Statistics and Mechanics, Beijing University of Technology, No. 100 Pingleyuan, 100124 Beijing, China; Institute of Statistics and Big Data, Renmin University of China, No. 59 Zhongguancun Street, 100872 Beijing, China

**Keywords:** Bayesian inference, cell typing, spatial pattern, single-cell spatial transcriptomics

## Abstract

The spatial transcriptomics is a rapidly evolving biological technology that simultaneously measures the gene expression profiles and the spatial locations of spots. With progressive advances, current spatial transcriptomic techniques can achieve the cellular or even the subcellular resolution, making it possible to explore the fine-grained spatial pattern of cell types within one tissue section. However, most existing cell spatial clustering methods require a correct specification of the cell type number, which is hard to determine in the practical exploratory data analysis. To address this issue, we present a nonparametric Bayesian model BACT to perform BAyesian Cell Typing by utilizing gene expression information and spatial coordinates of cells. BACT incorporates a nonparametric Potts prior to induce neighboring cells’ spatial dependency, and, more importantly, it can automatically learn the cell type number directly from the data without prespecification. Evaluations on three single-cell spatial transcriptomic datasets demonstrate the better performance of BACT than competing spatial cell typing methods. The R package and the user manual of BACT are publicly available at https://github.com/yinqiaoyan/BACT.

## Introduction

Uncovering spatial contents of one tissue can provide crucial insights into its spatial heterogeneity, thus elucidating how its cell types organize and work together to offer necessary functions. The spatial transcriptomics (ST) is a rapidly evolving biological technique that not only measures the gene expressions but also preserves the spatial location information for spots or cells in the tissue section [1, 2], so ST successfully avoids the spatial information loss in the single-cell RNA sequencing, which is caused by the tissue dissociation step.

Different ST techniques can provide ST data with different resolutions, which are often simply classified into two categories, spot level and single-cell level. On the one hand, for the previous, the 10x Genomics Visium platform [3] generates spot level ST data, where spots are arranged regularly in a square or triangle lattice and each spot contains a mix of cells, so it does not capture more subtle cell type information very well. On the other hand, ST technologies including MERFISH [4], STARmap [5], and Slide-seq [6] are able to measure gene expressions at or near the single-cell resolution, where the distribution of cells is irregular, so they can facilitate the understanding of the cell type spatial distribution in one tissue section. The focus of this paper is on spatial cell typing based on single-cell level ST data.

Several computational methods for spatial cell typing have been developed. FICT [7] first uses the cell locations to construct an undirected neighborhood graph, then combines a Gaussian gene expression model and a multinomial neighborhood model for joint likelihood derivation, and finally iteratively optimizes parameters and assigns cell classes via the EM algorithm. Li *et al*. [8] employs the graph neural networks to carry out unsupervised cell clustering, while SpaGCN [9] constructs a weight graph based on image pixel color channels and cells’ spatial information and then utilizes graph convolution networks for segmentation. STAGATE [10] relies on a graph attention auto-encoder to obtain low-dimensional latent embeddings of cells, which is followed by Louvain clustering.

Recently, a Bayesian hierarchical modeling framework BASS [11] is proposed, and its advantage is to simultaneously perform cell clustering and domain detection in the multi-scale and multi-sample analysis. Li *et al*. [12] provides a Bayesian mixture model, and it has the benefit to simultaneously clustering cells and selecting informative genes. BANKSY [13] has the ability to unify cell typing and tissue domain detection through the cell embedding in a product space of local neighborhood transcriptomics, and it is computationally scalable via building aggregates. However, all the methods mentioned above require a predetermined cell type number directly or indirectly, which may lead to unsatisfactory and inaccurate clustering performance if the cluster number is specified incorrectly. A recent statistical model BINRES [14] overcomes the cluster number prespecification using a nonparametric Bayesian prior, but its goal is region segmentation for spot-level and lattice-format ST data rather than cell typing. Therefore, there is an urgent need to resolve this issue for single-cell ST data.

To address this challenge, we develop a full Bayesian cell typing method BACT (BAyesian Cell Typing) to discover the complex spatial distributions of cell types for single-cell ST data. The motivation of our work stems from the rapid advances of ST techniques toward single-cell resolution, which necessitates the development of statistical methods tailored to the high-resolution ST data for cell type identification. Most existing approaches require a predetermination of the cell type number and may result in imprecise clustering outcomes if the provided cell type number is incorrect. BACT can explicitly use spatial information among cells to automatically learn the number of cell types from the data during the cell typing process. Specifically, in BACT, we first generalize the nonparametric Potts model [14, 15] that is only applicable to lattice-format ST data such that it can also adopt lattice-free single cell ST data, and we then carry out the Markov chain Monte Carlo algorithm for posterior inference. This nonparametric Bayesian prior can automatically identify the number of cell types, thus relaxing the limitation of most existing cell typing methods. We compare BACT against four state-of-the-art methods SpaGCN, STAGATE, BANKSY and BASS on three ST datasets collected by STARmap^*^, MERFISH, and Slide-seq, respectively. Through these applications to real-world datasets, BACT exhibits more accurate clustering performances as well as the capability of identifying rare cell types and learning the cell type number in a data-adaptive way, making it competitive with existing spatial cell typing methods.

## Materials and methods

### Data description

We apply BACT to three single-cell level ST datasets for the evaluation of its cell typing performance, and a spot level ST dataset is utilized to discuss the performance of BACT in the domain detection. The detailed information of these datasets are shown as follows:

- Mouse visual cortex STARmap^*^ data. The mouse visual cortex STARmap^*^ data were collected by Wang *et al*. [5]. The dataset comprises an ST count matrix, a cell coordinate matrix, and ground truth information for all the cells. The ST count matrix contains 1207 cells and 1020 genes. The cell type annotation used for quantitative comparison between all the methods is provided by Dong and Zhang [10].
- **Mouse hypothalamic preoptic region MERFISH data**. The mouse hypothalamic preoptic region MERFISH data consist of five tissue sections from the mouse hypothalamic preoptic region collected by Chen *et al*. [4]. Each of the five datasets consists of around 5500 cells and 155 genes. Notice that the raw data have been normalized. The MERFISH_0.19 is used for visualizing the cell typing performances of all methods, whose ST data matrix contains 5803 cells and 155 genes.
- **Mouse cerebellum Slide-seq data**. The mouse cerebellum Slide-seq data were collected by Rodriques *et al*. [6], and we use ‘Puck_180430_1.tar.gz’ to conduct the cell typing analysis. The ST count matrix comprises 24 847 cells and 18 906 genes, and we randomly selected 8000 cells for downstream analysis.
- **Human dorsolateral prefrontal cortex data**. The human dorsolateral prefrontal cortex data consist of twelve brain sections with manual annotations collected from three subjects [16]. The section 151507 is used for the analysis, whose raw count matrix contains 4226 spots and 33538 genes.

For all the real-world ST datasets, the ST data matrix is preprocessed by the R function ‘DataPreprocess’ in package BACT to generate the top 50 principal components (PCs), which are the input of the main R function ‘BACT’ in package BACT for model training. The cell/spot number, gene number, and download links of all the datasets are shown in Table 1.

**Table 1:** The cell/spot number, the gene number, and the download link of the publicly available real-world ST datasets.

ST dataset	Cell/Spot number	Gene number	Download link
STARmap^*^	1207	1020	Raw data: http://sdmbench.drai.cn/tcm/download/?file_path=/mnt/JINGD/data/file/sdmbench/db/STARmap_20180505_BY3_1k.h5ad Cell type annotation: https://drive.google.com/drive/folders/1I1nxheWlc2RXSdiv24dex3YRaEh780my?usp=sharing
MERFISH_0.04	5488	155	http://sdmbench.drai.cn/tcm/download/?file_path=/mnt/JINGD/data/file/sdmbench/db/MERFISH_0.04.h5ad
MERFISH_0.09	5557	155	http://sdmbench.drai.cn/tcm/download/?file_path=/mnt/JINGD/data/file/sdmbench/db/MERFISH_0.09.h5ad
MERFISH_0.14	5926	155	http://sdmbench.drai.cn/tcm/download/?file_path=/mnt/JINGD/data/file/sdmbench/db/MERFISH_0.14.h5ad
MERFISH_0.19	5803	155	http://sdmbench.drai.cn/tcm/download/?file_path=/mnt/JINGD/data/file/sdmbench/db/MERFISH_0.19.h5ad
MERFISH_0.24	5543	155	http://sdmbench.drai.cn/tcm/download/?file_path=/mnt/JINGD/data/file/sdmbench/db/MERFISH_0.24.h5ad
Slide-seq	24 847	18 906	https://singlecell.broadinstitute.org/single_cell/study/SCP354/slide-seq-study∖#study-download. Download the barcode file ‘Puck_180430_1.tar.gz’
DLPFC section 151507	4226	33 538	http://sdmbench.drai.cn/tcm/download/?file_path=/mnt/JINGD/data/file/sdmbench/db/151507.h5ad

### Model setup

BACT is motivated by the previously proposed Bayesian region segmentation method BINRES [14] that performs clustering only for the spot-level lattice-format ST data (e.g. 10x Genomics Visium). To relax this constraint, in the nonparametric Potts model [15], we need to change its neighborhood selection rule to allow for lattice-free single-cell ST data. Specifically, in BINRES, two spots are neighbors if they are directly connected without any intermediate spot in a lattice, while in BACT for two cells labeled by indices $i$ and $j$  $(i\neq j)$, cell $j$ is one neighbor of cell $i$ if cell $j$ is one of the $k$ nearest neighbors of cell $i$, where the integer $k$ needs to be specified by users. This relaxation allows for the application of the nonparametric Potts model to single-cell ST data.

We assume there are $n$ cells, and denote the set of $k$ nearest neighbors of cell $i$ by $\mathrm{Nei}_{k}(i)$, which does not include cell $i$ itself. Cell $j$ is one neighbor of cell $i$ if $j \in \mathrm{Nei}_{k}(i)$. The indicator $C_{i}$ of cell $i$ is the cell type to which cell $i$ belongs. We use $\mathbf{C}$ to be the indicator vector $(C_{1}, C_{2}, \ldots , C_{n})$. The nonparametric Potts model using the $k$ nearest neighbor selection rule becomes the following joint probabilistic distribution for the cell type indicator vector $\mathbf{C}$: 


\begin{align*} \mathbb{P}&(\mathbf{C}|\beta, \{\pi_\ell\}_{\ell=1}^{\infty}) =\frac{\prod_{i=1}^{n}\pi_{C_{i}}\cdot \exp\left\{\beta\sum_{i=1}^{n}\sum_{j \in \mathrm{Nei}_{k}(i)}\mathbb{I}(C_{i} = C_{j})\right\}}{\sum_{\mathbf{C}\in \mathbb{N}_{+}^{n}} \prod_{i=1}^{n}\pi_{C_{i}}\cdot\exp\left\{\beta\sum_{i=1}^{n}\sum_{j \in \mathrm{Nei}_{k}(i)}\mathbb{I}(C_{i} = C_{j})\right\}}. \nonumber\end{align*}

The spatial interaction parameter $\beta $ is positive and captures the spatial dependence strength between neighboring spots. We denote by $\{\pi _{\ell }\}_{\ell =1}^{\infty }$ the external field term parameters in the nonparametric Potts model [15], representing the probability of one cell belonging to certain cell type when the spatial interaction effects between neighboring cells are not considered. The parameters satisfy $\pi _{\ell }>0$ and $\sum _{\ell =1}^{\infty }\pi _{\ell } = 1$. $\mathbb{N}_{+}^{n}$ is the $n$-fold Cartesian product of the positive integer set $\mathbb{N}_{+}$.

**Figure 1:**
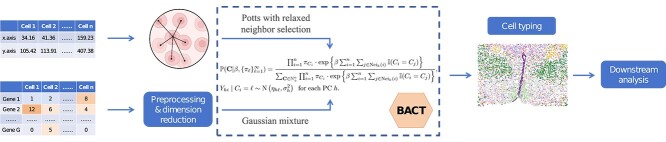
The schematic of BACT.

A data preprocessing procedure is required before implementing BACT, and it involves normalizing the raw gene count data and applying a logarithmic transformation. Following Li *et al*. [11], we utilize the principal component analysis to reduce dimensionality and extract the top PCs as the input of BACT. We note that other dimension reduction algorithms can be used here to replace PCs. We further denote the selected $H$ top PCs for spot $i$ by $\mathbf{Y}_{i} = (Y_{1i},\ldots ,Y_{Hi})^\top $, and we assign a multivariate normal distribution with a diagonal covariance matrix to $\mathbf{Y}_{i}$ conditional on the cell type indicator $C_{i}$, that is, $Y_{hi} \mid C_{i}=\ell $  $\sim \mathrm{N}\left (\eta _{h \ell }, \sigma _{h}^{2}\right )$ for each component $h$  $(h=1,\ldots , H)$, where $\eta _{h \ell }$ and $\sigma _{h}^{2}$ denote the mean and variance of $Y_{hi}$, respectively. Figure 1 demonstrates the schematic of BACT.

In the Bayesian framework, we assign priors to the parameters of the BACT model. Following the prior specification scheme in Yan and Luo [14], a truncated normal prior $\mathrm{N}(a_{\beta }, \tau ^{2}_{\beta })\mathbb{I}(\beta> 0)$ is designated for the spatial interaction parameter $\beta $. The prior for the mean value $\eta _{h\ell }$ of PC $h$ is specified as a normal distribution $\mathrm{N}(a_{\eta }, b_{\eta }^{2})$, and an inverse gamma distribution inv-$\Gamma (\kappa , \tau )$ is assigned to the variance $\sigma _{h}^{2}$ of PC $h$ with shape parameter $\kappa $ and scale parameter $\tau $. We assign the stick-breaking process prior [17] to the parameters $\{\pi _\ell \}_{\ell =1}^{\infty }$, ensuring that $\pi _{\ell }>0$ and $\sum _{\ell =1}^{\infty }\pi _\ell =1$ almost surely. Specifically, we construct $\{\pi _\ell \}_{\ell =1}^{\infty }$ via independent and identically distributed variables $\{\xi _\ell \}_{\ell =1}^{\infty }$ generated from a beta distribution $\text{Beta}(1,\alpha )$ such that $\pi _{1}=\xi _{1}$ and $\pi _\ell = \xi _\ell \prod _{m=1}^{\ell -1}(1-\xi _{m})$ for $\ell \ge 2$. $\alpha $ is referred to as the concentration parameter, and when there is no spatial interaction a higher value of $\alpha $ tends to giving a larger cluster number.

### Bayesian inference

Subsequently, we carefully design a Markov chain Monte Carlo method to perform the statistical inference of BACT. The cell type indicators $C_{i}$ have the characteristic of taking all positive integer values, which poses computational challenges in the sampling procedure. To overcome this difficulty, we employ a slice sampling scheme [18] and introduce auxiliary variables $\mathbf{u}=\{u_{i}:i=1,\ldots ,n\}$ by independently drawing them from a uniform distribution on $(0,1)$, which results in the augmented conditional prior distribution $\mathbb{P}(\mathbf{C}, \mathbf{u}\;|\;\beta ,$  $ \{\pi _\ell \}_{\ell =1}^{\infty }) $  $ \propto \prod _{i=1}^{n}\mathbb{I}(u_{i}\leq \pi _{C_{i}})\times \exp \left \{\beta \sum _{i=1}^{n}\sum _{j \in \mathrm{Nei}_{k}(i)}\mathbb{I}(C_{i} = C_{j})\right \}$. Since the summation of $\{\pi _\ell \}_{\ell =1}^{\infty }$ equals one, the candidate sets $\{\ell : u_{i} \leq \pi _\ell \}$ contains only a finite number of elements, making it straightforward to sample an updated $C_{i}$ from this set. Finally, the partially collapsed Gibbs sampler [19] for posterior inference is shown as follows, where the symbol ‘−’ represents ‘given all other variables and data’, and a workflow of the posterior sampling algorithm is demonstrated in Supplementary Section S1.

(a) Update $\beta $ and $\{\xi _\ell \}_{\ell =1}^{\infty }$ simultaneously from their conditional distribution shown as follows:
  \begin{align*} f\left( \beta, \{\xi_\ell\}_{\ell=1}^{\infty} | - \right) \propto \ & \frac{\prod_{i=1}^n\pi_{C_{i}}\cdot \exp\left\{\beta\sum_{i=1}^n\sum_{j \in \mathrm{Nei}_k(i)}\mathbb{I}(C_{i} = C_{j})\right\}}{\mathcal{C}(\beta, \{\xi_\ell\}_{\ell=1}^{\infty} )} \\ &\times \mathrm{N}\left(\beta \mid a_{\beta}, \tau_\beta^{2}\right) \mathbb{I}(\beta>0) \times \prod_{\ell=1}^{\infty}\text{Beta}(\xi_\ell | 1, \alpha). \end{align*}
  The normalizing constant $\mathcal{C}(\beta , \{\xi _\ell \}_{\ell =1}^{\infty } )$ equals $\sum _{\mathbf{C}\in \mathbb{N}_{+}^{n}} \prod _{i=1}^{n}\pi _{C_{i}}\cdot \exp \left \{\beta \sum _{i=1}^{n}\sum _{j \in \mathrm{Nei}_{k}(i)}\mathbb{I}(C_{i} = C_{j})\right \}$.
(b) Sample the auxiliary variables $\{u_{i}\}$ from the uniform distribution $\text{Unif}(0, \pi _{C_{i}}).$
(c) Update the cell type indicators $C_{i}$  $(i=1,\ldots ,n)$ in a sequential manner from the following full conditional distribution:
  \begin{align*} & \begin{aligned} \mathbb{P}(C_{i} = \ell \mid -) \propto \exp \left(\beta \sum_{j \in \mathrm{Nei}_k(i)} \mathbb{I}\left(C_{j}=\ell\right)\right)\times \prod_{h=1}^H \mathrm{N}\left(Y_{hi}\mid \eta_{h \ell}, \sigma_{h}^{2}\right). \end{aligned} \end{align*}
  The positive integer $\ell $ takes values in the finite set $\{\ell : \pi _{\ell } \ge u_{i}\}$.
(d) For each PC $h$, update $\eta _{h \ell }$ from a normal distribution $\mathrm{N}(\widetilde a_{\eta , h \ell }, \widetilde b_{\eta , h \ell }^{2})$ with $\widetilde a_{\eta , h \ell } = \frac{\sum _{i: C_{i}=\ell }Y_{hi} / \sigma _{h}^{2}+a_{\eta } / b_{\eta }^{2}}{m_{\ell } / \sigma _{h}^{2}+1 / b_{\eta }^{2}}$ and $\widetilde b_{\eta , h \ell }^{2} = \frac 1{m_{\ell } / \sigma _{h}^{2}+1 / b_{\eta }^{2}}$. Here $m_{\ell }= \sum _{i=1}^{n} \mathbb{I}(C_{i} = \ell )$ is the number of elements in the cell type $\ell $.
(e) The PC-specific variance $\sigma _{h}^{2}$ is sampled from an inverse gamma distribution $\text{inv-}\Gamma \left (\widetilde \kappa , \widetilde \tau \right )$ with shape $\widetilde \kappa = \kappa + n/2$ and scale $\widetilde \tau =\tau +\frac{1}{2} \sum _{i=1}^{n}(Y_{hi}-\eta _{hC_{i}})^{2}$.

Notice that for Step (a), a standard Gibbs sampling is hard to implement because the normalizing constant $\mathcal{C}(\beta , \{\xi _\ell \}_{\ell =1}^{\infty } )$ is computationally intractable. Therefore, we apply the double Metropolis–Hastings method [20, 21] to address this problem and decompose step (a) into three substeps as follows:

(a1) Generate proposals $(\{\xi ^{*}_\ell \}_{\ell =1}^{\infty }, \beta ^{*})$. We propose $\{\xi _\ell ^{*}\}_{\ell =1}^{\infty }$ and $\beta ^{*}$, respectively, from $q(\cdot |\beta ) \times \prod _{\ell \geq 1}q(\cdot | \xi _\ell )$ given their current values, where
  \begin{align*} & \begin{aligned} &q(\xi_\ell^* \mid \xi_\ell)=\left\{ \!\!\begin{aligned} &\mathrm{TrunNormal}(\xi_\ell^*\;|\;\xi_\ell, \tau_{0}^2, 0, 1), &&\!\! \ell = 1,...,R_0 \\ &\text{Beta}(\xi_\ell^*\;|\;1, \alpha), && \!\!\ell = R_0 \!+\! 1, R_0 \!+\! 2,... \end{aligned} \right.\\ &q(\beta^{*} \mid \beta) = \mathrm{TrunNormal}(\beta^{*}\;|\;\beta, \tau_1^2, 0, +\infty). \end{aligned} \end{align*}
  The term $R_{0}$ is a fixed and relatively large positive integer. We denote by TrunNormal$(\cdot \;|\;\mu , \sigma ^{2}, a,b)$ the probability density function of the normal distribution $\mathrm{N}(\mu , \sigma ^{2})$ truncated on the interval $(a,b)$, where $-\infty \le a < b \le + \infty $. It is evident that, following the construction rule in the stick-breaking process, $\{\pi ^{*}_\ell \}_{\ell =1}^{\infty }$ generated by the proposals $\{\xi ^{*}_\ell \}_{\ell =1}^{\infty }$ continues to satisfy $\pi ^{*}_\ell>0$ and $\sum _{\ell =1}^{\infty }\pi ^{*}_\ell = 1$ with probability one.
(a2) Generate auxiliary variables $\mathbf{C}^{*}$. Initially, we set the auxiliary variables $\mathbf{C}^{*}$ to its current value $\mathbf{C}$, and sequentially simulate each element $C^{*}_{i}\ (1\leq i\leq n)$ from the distribution $\mathbb{P}(C_{i}^{*} \mid \beta ^{*}, \{\pi ^{*}_\ell \}_{\ell =1}^{\infty }, \mathbf{C}^{*}_{-i})$. The notation $\mathbf{C}^{*}_{-i}$ denotes the vector $\mathbf{C}^{*}$ with the $i$th element removed. $C^{*}_{i}$ is then assigned to the positive integer $\ell $ with probability $p^{\ast }_{i\ell }$ based on the proposals $\{\xi _\ell ^{*}\}_{\ell =1}^{\infty }$ and $\beta ^{*}$,
  \begin{align*} &\!\!\!\!\!\! p^{\ast}_{i\ell} = \left\{ \begin{aligned} &\frac{\pi^*_\ell \exp\{\beta^* \sum_{j \in \text{Nei}_k(i)} \mathbb{I}(C^*_j = \ell)\}} { 1 + \sum_{\ell=1}^{R_i} \pi^*_\ell \left[ \exp\{\beta^* \sum_{j \in \text{Nei}_k(i)} \mathbb{I}(C^*_j = \ell)\} - 1 \right]} &&\!\!\! \mathrm{for}\;\; \ell \le R_i \\ &\frac{\pi^*_\ell} { 1 + \sum_{\ell=1}^{R_i} \pi^*_\ell \left[ \exp\{\beta^* \sum_{j \in \text{Nei}_k(i)} \mathbb{I}(C^*_j = \ell)\} - 1 \right]} &&\!\!\! \mathrm{for}\;\; \ell> R_i \end{aligned}\right.. \end{align*}
  The term $R_{i}=\max _{j \in \mathrm{Nei}_{k}(i)}C^{*}_{j}$ represents the maximal value of the auxiliary variables among the $k$ neighbors of cell $i$. We employ the inverse cumulative distribution function method to update $C_{i}^{*}$. Specifically, we generate a uniform variable $v_{i}$ from $\text{Unif}(0,1)$. If $v_{i} \le p^{\ast }_{i1}$ then we set $C_{i}^{*}$ to one. Otherwise, we need to determine $\ell $ such that $\sum _{j=1}^{\ell -1} p^{\ast }_{ij} < v_{i} \le \sum _{j=1}^{\ell } p^{\ast }_{ij}$ and then assign $C_{i}^{*}$ to $\ell $.
(a3) Accept or reject the proposals $(\{\xi ^{*}_\ell \}_{\ell =1}^{\infty }, \beta ^{*})$. After obtaining the auxiliary variables $\mathbf{C}^{*}$, we would accept the proposals $\{\xi ^{*}_\ell \}_{\ell =1}^{\infty }$ and $\beta ^{*}$ with probability $\min (r, 1)$, where
  \begin{align*} \qquad r =& \frac{ \prod_{\ell=1}^{R_{0}} (1- \xi_\ell^{*})^{\alpha - 1} \cdot\mathrm{TrunNormal}(\beta^{*} | a_\beta, b_\beta^{2}, 0, +\infty) } { \prod_{\ell=1}^{R_{0}} (1- \xi_\ell)^{\alpha - 1} \cdot \mathrm{TrunNormal}(\beta | a_\beta, b_\beta^{2}, 0, +\infty) } \times \frac{ \prod_{\ell=1}^{R_{0}} \mathrm{TrunNormal}(\xi_\ell | \xi_\ell^{*}, \tau_{0}^{2},0,1) \cdot \mathrm{TrunNormal}(\beta | \beta^{*}, \tau_{1}^{2},0, +\infty) }{ \prod_{\ell=1}^{R_{0}}\mathrm{TrunNormal}(\xi_\ell^{*} | \xi_\ell, \tau_{0}^{2},0,1) \cdot \mathrm{TrunNormal}(\beta^{*} | \beta, \tau_{1}^{2},0, +\infty) } \nonumber\\ & \times \frac{ \prod_{i=1}^{n} \pi_{C_{i}}^{*} \exp\left( \beta^{*} \sum_{i=1}^{n}\sum_{j \in \mathrm{Nei}_{k}(i)} \mathbb{I}(C_{i} = C_{j}) \right) \cdot \prod_{i=1}^{n} \pi_{C_{i}^{*}} \exp\left( \beta \sum_{i=1}^{n}\sum_{j \in \mathrm{Nei}_{k}(i)} \mathbb{I}(C_{i}^{*} = C_{j}^{*}) \right) }{ \prod_{i=1}^{n} \pi_{C_{i}} \exp\left( \beta \sum_{i=1}^{n}\sum_{j \in \mathrm{Nei}_{k}(i)} \mathbb{I}(C_{i} = C_{j}) \right) \cdot \prod_{i=1}^{n} \pi_{C_{i}^{*}}^{*} \exp\left( \beta^{*} \sum_{i=1}^{n}\sum_{j \in \mathrm{Nei}_{k}(i)} \mathbb{I}(C_{i}^{*} = C_{j}^{*}) \right) }. \nonumber\end{align*}

### Implementation summary of BACT and competing methods

This subsection delineates the implementation summaries of all methods as outlined below, and more details can be found in Supplementary Section S2.

- BACT: For BACT, we set the initial cell cluster number as the number of underlying ground truth provided by Yuan *et al*. [22] for the STARmap^*^ and MERFISH datasets, and as the number of cell layers provided by Singhal *et al*. [13] for the Slide-seq dataset, aiming to prevent overfitting that may arise from a large initial cluster number and to facilitate a more effective estimation of the heterogeneous gene expression profiles across different cell types. We note that the initial cell cluster number only serves as initialization and is not necessarily equal to the final estimated cluster number. The number of neighbors is set to six and the number of PCs is set to 50 in all the real applications. The remaining parameters are set to their default values.
- SpaGCN: SpaGCN is a cell typing algorithm based on graph convolutional network [9], and its Python implementation code is publicly accessible via GitHub https://github.com/jianhuupenn/SpaGCN. Since the original code includes preprocessing steps for raw count data, we made a few adjustments to enable the updated code to be directly applied to the normalized gene expression data (for example, the MERFISH dataset). We set the prespecified clustering number as the number of annotated cell types. Since the datasets used in the manuscript do not contain image data, we set the argument ‘img’ to zero. The other parameters were maintained at their default settings.
- STAGATE: STAGATE is an approach for cell typing that employs graph attention autoencoders to derive low-dimensional latent embeddings of cells [10]. The Python code for STAGATE can be accessed publicly on GitHub https://github.com/zhanglabtools/STAGATE. Normalization and logarithmic transformation of the raw data matrix are performed before users implement the main function ‘train_STAGATE’. Following the guidelines provided by Dong and Zhang [10], we did not incorporate the cell-type-aware spatial neighbor network by setting the alpha parameter to its default value of zero. The spatial network was constructed by selecting $k$ nearest neighbors of each cell with $k=6$. We assigned the predefined number of clusters to the annotated cell type number. All other parameters are maintained at their default settings. For cell clustering, STAGATE utilizes the ‘mclust_R’ function based on the R package mclust.
- BANKSY: BANKSY performs cell typing through the cell embedding in a product space of local neighborhood transcriptomics [13]. The Python code of BANKSY is publicly available on GitHub https://github.com/prabhakarlab/Banksy_py. Initialization is conducted by the function ‘initialize_banksy’ based on the $k$ nearest neighbors (argument: k_geom) before implementing the model. The number of neighbors is set to six and the number of PCs is set to 50 in all the real applications. We set the prespecified clustering number as the number of annotated cell types provided by the cell type annotation information. The remaining parameters are set to their default values. We employs ‘mclust_R’ as the clustering algorithm for BANKSY using R package mclust.
- BASS: BASS is able to perform cell type identification and domain detection simultaneously with multiple tissue sections [11]. The R code of BASS is publicly available on GitHub https://github.com/zhengli09/BASS. Specifically, we first create a BASS object by the function ‘createBASSObject’ with the number of neighbors set to six (argument: k) and the numbers of cell types (argument: C) and regions (argument: R) determined according to the annotation of the data. We then preprocess the raw data by the function ‘BASS.preprocess’ via the principal component analysis and obtain the top 50 PCs (argument: nPC). The remaining parameters are set to their default values.

## Results

To assess the cell typing performance of BACT, we compare it to four state-of-the-art methods SpaGCN [9], STAGATE [10], BANKSY [13], and BASS [11] on three single-cell level ST datasets. We also show the domain detection ability of BACT on a spot level ST dataset. We quantitatively compared BACT with other competing methods via the adjusted Rand index (ARI) [23] that is one of the commonly used clustering evaluation metric measuring the similarity between two data partitions.

### Mouse visual cortex STARmap^*^ data

**Figure 2:**
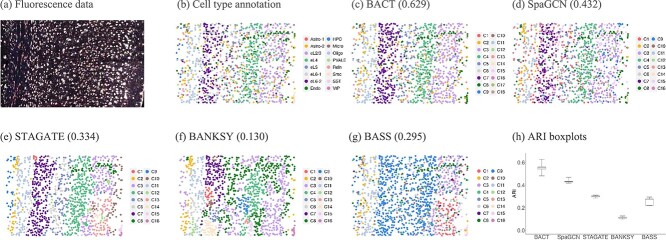
(a) Fluorescence data of STARmap provided by Wang *et al*. [5]. (b) Underlying cell type annotation for the mouse visual cortex STARmap^*^ data. The cell typing performances are shown for (c) BACT, (d) SpaGCN, (e) STAGATE, (f) BANKSY, and (g) BASS, where the number in the parentheses is the corresponding ARI value. (h) The ARI boxplots to quantitatively compare the cell type identification accuracy based on five random repeats of all the methods. The medians for BACT, SpaGCN, STAGATE, BANKSY, and BASS are 0.553, 0.432, 0.302, 0.111, and 0.277, respectively. The boxplots exhibit medians, interquartile ranges, and maximal and minimal values throughout the paper, represented by horizontal line in each box, boxes, and whiskers, respectively.

STARmap is an imaging-based ST technique that enables the single-cell resolution detection of gene expressions in mouse visual cortex tissue samples [5], and STARmap^*^ is an extension of STARmap to include a larger set of target genes and cover a larger spatial domain. The raw fluorescence image data of STARmap^*^ is shown in Fig. 2(a) provided by Wang *et al*. [5].

We conducted 6000 iterations to implement BACT, with the first 3000 as burn-in. The execution time was about 13.5 s per 100 iterations on a MacBook Pro with Intel Core i5 CPU at 2,GHz and 16,GB of RAM. In Fig. 2(c), BACT detected 18 distinct cell types and achieved the best cell typing performance with ARI=0.629 in comparison to the cell type annotation (Fig. 2(b)). This indicated that it was able to unveil the underlying cell heterogeneity in single-cell ST data. Specifically, BACT accurately identified the annotated cell types eL2/3, eL4, eL6-2, and Oligo as the estimated clusters C3, C4, C7, and C11, respectively. These cell types exhibited laminar spatial patterns and clear boundaries. Moreover, BACT also effectively captured cell types such as Astro-2 and Endo, which illustrated irregular distribution pattern throughout the tissue section. The further analysis of the additional cell types detected by BACT is detailed in Supplementary Section S3.

The cell typing results of SpaGCN, STAGATE, BANKSY, and BASS were shown in Fig. 2(d)–(g) with the prespecified cell type number being 16 based on the cell type annotation, and the computational time of them was 13.7 s per 100 iterations, 9.3 s per 100 iterations, 14.2 s in total (since BANKSY is not a iterative method), and 2.9 s per 100 iterations, respectively. Figure 2(c)–(g) showed that the cell clustering accuracy of the competing methods was lower than BACT in terms of ARI. SpaGCN (ARI=0.432) also identified cell types with regular shapes, such as eL2/3, eL4, eL6-2 and Oligo. However, SpaGCN did not fully capture cell types like Astro-2, which are distributed irregularly throughout the tissue section, as SpaGCN tended to partition cells into a multi-layer structure. The ARI of STAGATE (ARI=0.334) was slightly lower than that of SpaGCN. On the one hand, STAGATE incorrectly merged these cell types into the estimated cell type C10 on the right side of the tissue section, indicating that STAGATE may not be good at detecting cell types with a low cell number, thus leading to a lower ARI. On the other hand, Astro-2 and Endo were irregularly distributed across the entire spatial domain of the tissue section, which do not exhibit clear layer structures. STAGATE tended to merge them into nearby layer regions, thereby reducing the accuracy of cell typing. BANKSY obtained the lowest ARI of 0.130. It did not accurately identify the cell types such as eL2/3, eL4, eL6-2, and Oligo. Although BASS (ARI=0.295) successfully identified Endo, it incorrectly merged eL4, eL5, eL6-1, and eL6-2 into C9. To guarantee the stability of each method, we randomly repeated all methods for five times, and the ARI boxplots were shown in Fig. 2(h). BACT outperformed other methods over the five repeats in terms of ARI, demonstrating its ability to identify complex cell type patterns.

Moreover, we selected three different settings of parameters for the competing methods, each repeated five times, and provided the results in Supplementary Section S4. The boxplots shown in Supplementary Fig. S3 demonstrate that their ARI values under different parameter settings are similar, so the cell typing results given by these methods are consistent. To test whether the cell typing performances of the competing methods can be enhanced using the cell type number detected by BACT, we added another experiment in the analysis of the STARmap^*^ data in Supplementary Section S5.

After carefully investigating the BANKSY paper [13], we noticed that BANKSY focused on the domain detection of the STARmap^*^ dataset and provided domain structure annotations of the tissue section. In contrast, our study concentrated on the cell type identification of single cell ST data, and for all the methods, we compared the cell clustering results to the cell type annotation rather than domain structure. Therefore, the ARI results in our study differed from those in the BANKSY paper.

### Mouse hypothalamic preoptic region MERFISH data

**Figure 3:**
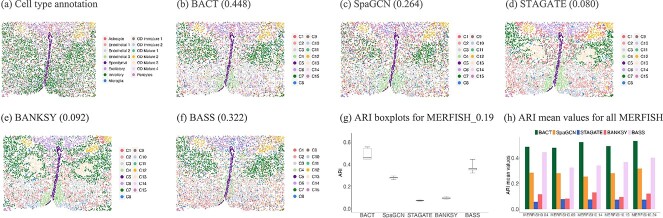
(a) Underlying cell type annotation for the mouse hypothalamic preoptic region MERFISH_0.19 data. The cell typing performances are shown for (b) BACT, (c) SpaGCN, (d) STAGATE, (e) BANKSY, and (f) BASS, where the number in the parentheses is the corresponding ARI value that applies to other panels. (g) The ARI boxplots for MERFISH_0.19 data, where the medians for BACT, SpaGCN, STAGATE, BANKSY, and BASS are 0.460, 0.281, 0.075, 0.092, and 0.362, respectively. (h) The bar plots illustrating each method’s mean ARI values across random repeats for all MERFISH sections.

This dataset consists of five tissue sections from the mouse hypothalamic preoptic region collected by Chen *et al*. [4]. The authors leveraged the single-cell transcriptome imaging method MERFISH to simultaneously capture the gene expressions and spatial locations *in situ* with high specificity and accuracy. Each section consists of around 5500 cells and 155 genes. We used the MERFISH_0.19 section to carry out the cell typing analysis, and the annotated cell types were provided in Fig. 3(a). Among the cell typing methods, BACT obtained the highest ARI value with 0.448 (Fig. 3(b)), and BASS ranked the second with ARI=0.322 (Fig. 3(f)). The results of SpaGCN, STAGATE, and BANKSY were displayed in Fig. 3(c)-(e), and their ARI values were less than 0.3. The ARI boxplots for all methods were depicted in Fig. 3(g), based on five independent random repeats for each method. BACT outperformed other methods owing to its capability to discern subtle cell types. We also provided the bar plots of ARI mean values using the five MERFISH sections in Fig. 3(h). The specific cell typing results for the other four sections as well as corresponding ARI boxplots are provided in Supplementary Fig. S1.

### Mouse cerebellum Slide-seq data

**Figure 4:**
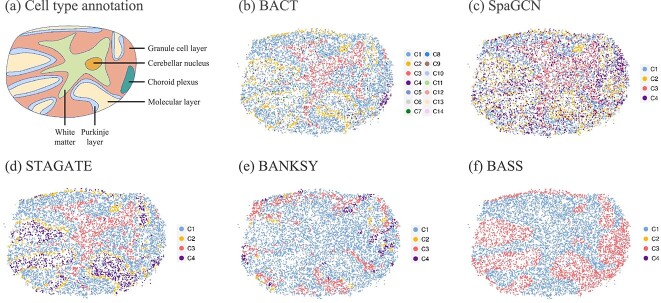
(a) Schematic diagram of the cell type annotation for the Slide-seq tissue section. The cell typing performances of (b) BACT, (c) SpaGCN, (d) STAGATE, (e) BANKSY, and (f) BASS for mouse cerebellum Slide-seq data.

The mouse cerebellum is an essential structure of the mouse brain and regulates motor functions including motor coordination, balance, and learning. Rodriques *et al*. [6] applied Slide-seq technique to profile gene expressions at the single-cell resolution in mouse cerebellum tissue samples. We used the ‘Puck_180430_1’ dataset for subsequent analysis. The cell coordinates and the raw count data matrix are stored in the BeadLocationsForR.csv and MappedDGEForR.csv files, respectively, where the count matrix contains 24,847 cells and 18,906 genes. Since the cell type annotation is absent in the Slide-seq dataset, we utilized the biological information provided by Singhal *et al*. [13], Shang and Zhou [24] and the Allen Brain Atlas platform [25], and plotted a schematic diagram (Fig. 4(a)) based on Fig. 3(a) of Shang and Zhou [24]. The prespecified cell type number of the competing methods is set to four according to Singhal *et al*. [13].

Figure 4(b)–(f) illustrated the efficacy of cell-type clustering by BACT, SpaGCN, STAGATE, BANKSY, and BASS. Notice that each method was executed five times randomly, and a representative result was selected for display in this figure. The specific cell typing results of all methods based on the five random repeats were provided in Supplementary Fig. S2. BACT successfully identified 14 distinct cell types, where C1–C3 represent the granular layer, the purkinje neurons layer, and the white matter, respectively. With respect to the molecular layer, cell types C5–C14 were associated with this domain, implying that molecular layer interneurons might comprise multiple cell subtypes with diverse gene expression profiles, indicating a variety of complex functionalities in this mouse brain domain. Moreover, BACT also uncovered the cell cluster C4, which was not accurately recognized by SpaGCN, STAGATE, BANKSY, and BASS.

Furthermore, we observed that the cell cluster C4 detected by BACT corresponded to a distinct functional area in the mouse brain. We first conducted the gene differential expression (DE) analysis using the R package edgeR to find DE genes of cell cluster C4 with a false discovery rate below 0.01. The names of the DE genes were given in Table 2. Subsequently, we uploaded the 15 DE genes to the Gene Set Enrichment Analysis platform using the gene ontology dataset, which is available at the Broad Institute website http://software.broadinstitute.org/gsea/msigdb/annotate.jsp. We found 30 significant pathways, and they were reported in Supplementary Table S1. It revealed that these DE genes were enriched with the ‘GOCC_EXTRACELLULAR_SPACE’ pathway that involves the storage and transfer of molecules and structural substances outside the cell, so this observation may establish an association between the cell cluster C4 and the choroid plexus, a brain structure closely related to the extracellular environment and primarily responsible for the production and regulation of cerebrospinal fluid. Therefore, the detection of cell cluster C4 revealed the capacity of BACT to identify heterogeneous cell types and discover fine-grained functional structures in this dataset.

**Table 2:** Differentially expressed gene names detected by the R package edgeR based on the cell typing result of BACT in the mouse cerebellum Slide-seq data analysis.

Differentially expressed genes	
*Calml4*	*Calm2*	*Enpp2*	*Folr1*	*F5*	*Gabra6*	*Igfbp2*	*Malat1*
*Kcne2*	*Kcnj13*	*Ptgds*	*Rbp1*	*Snap25*	*Ttr*	*1500015O10Rik*	

We also carried out the sensitivity analysis by changing the value of the hyperparameter $a_\beta $ to investigate the effects of stronger prior spatial interactions on the cell typing performance. A comprehensive discussion of this investigation is provided in Supplementary Section S6.

### Human dorsolateral prefrontal cortex data

BACT is designed for single-cell ST data, but as a statistical clustering tool, it can be applied to spot-level ST data in principle, such as the 10x Visium DLPFC dataset. However, as the primary task for the spot-level data is to uncover domain structures rather than to identify cell types, it is expected that BACT may not perform as well as those methods tailored for domain detection. Detailed experimental results of the models applied to DLPFC section 151507 are provided in Supplementary Section S7.

## Discussion

BACT is a nonparametric Bayesian statistical method designed for the cell typing analysis of single-cell ST data. The novelty of BACT attributes to the utilization of the nonparametric Potts model with a generalized neighborhood selection rule, so BACT enjoys the advantage of explicit modeling of the spatial dependence among neighboring cells. It also has the flexibility of cell type identification in the tissue section without the need for a predetermined cell type number. Moreover, BACT improves the interpretation over neural network-based methods in the following two main aspects. First, BACT directly models the latent data generating process and can incorporate expert knowledge or existing information into the prior distributions, thereby capturing the underlying mechanisms of single-cell ST data rather than deriving latent embeddings of data through statistically less interpretable neural networks. Second, BACT utilizes the well-studied nonparametric Potts model to explicitly capture the spatial dependence pattern of ST data and automatically learns the number of cell types from the data.

We evaluated the performance of BACT for cell typing on three single-cell ST datasets—STARmap^*^, MERFISH, and Slide-seq. Overall, BACT demonstrates better performance in identifying cell types than other state-of-the-art methods. In our experiments, the datasets selected to exhibit the cell typing results of the methods were not cherry-picked. Instead, they are commonly used in the field of ST. BACT consistently demonstrates good performances of cell typing across these datasets, supporting its generalization ability to broader applications. Therefore, we envision that BACT will be a powerful Bayesian solution to the unsupervised spatial cell typing problem of single-cell ST data.

There are still several directions to improve the effectiveness of BACT. First, BACT uses the principal components of gene expressions in dimension reduction and clustering analysis, which may result in the information loss of the original gene expressions. To tackle this issue, we will consider modeling the original raw count data directly and take into account the zero inflations in single-cell data. Second, although BACT outperforms the competing methods in cell typing, its execution time is relatively longer than other methods due to the iterative sampling scheme by the Markov chain Monte Carlo algorithm. Therefore, to reduce the computational burden, we may resort to the variational Bayes approaches [26, 27] with fast optimization algorithms.

**Key Points**

- BACT is a novel nonparametric Bayesian model designed to perform cell typing for single-cell spatial transcriptomics data based on the gene expressions and spatial coordinates.
- BACT utilizes the nonparametric Potts prior to build the spatial dependency of neighboring cells and has the capability to learn the cell type number in a data-driven way.
- BACT exhibits more accurate clustering performances than competing approaches as well as the capability of identifying rare cell types, making it competitive with existing spatial cell typing methods.

## Supplementary Material

BACT_supplementary_bbae689

## Code availability

The source code that can reproduce the results in this paper is at https://github.com/yinqiaoyan/BACT_code_data. The R package and the user manual of BACT are publicly available at https://github.com/yinqiaoyan/BACT.
