# Supplementary material for: BACT: nonparametric Bayesian cell typing for single-cell spatial transcriptomics data
Source: Brief Bioinform. 2025 Jan 3;26(1):bbae689. doi: 10.1093/bib/bbae689 (PMC11697130; doi:10.1093/bib/bbae689)
Supplement: BACT_supplementary_bbae689 [file bact_supplementary_bbae689.pdf]

Supplementary Materials for BACT: nonparametric Bayesian cell typing for single-cell spatial transcriptomics data

Yinqiao Yan[1] and Xiangyu Luo[2,*]

[1]School of Mathematics, Statistics and Mechanics, Beijing University of Technology

[2]Institute of Statistics and Big Data, Renmin University of China

[*]Corresponding author, xiangyuluo@ruc.edu.cn

S1 Workflow of the posterior sampling procedure

For the posterior inference of BACT, we design a Markov chain Monte Carlo method to iteratively update the parameters, and adopt the partially collapsed Gibbs scheme (Van Dyk and Park, 2008) to improve the efficiency of the sampling method. To facilitate a better understanding and clear visualization of the working flow, we offer an outline of the posterior sampling algorithm as follows.

Algorithm 1 Partially collapsed Gibbs scheme to carry out posterior sampling

Initialization: Spatial interaction parameter $\beta^{(0)}$, Cell-type-specific weights $\{\pi_\ell^{(0)}\}_{\ell=1}^{\infty}$, Auxiliary variables $\{u_i^{(0)}\}_{i=1}^{n}$, Cell type indicators $\{C_i^{(0)}\}_{i=1}^{n}$, Gene expression means $\{\eta_{h\ell}^{(0)}\}_{h=1}^{H}$, Gene expression variances $\{\sigma_h^{2(0)}\}_{h=1}^{H}$, Number of burn-in steps b, Number of total sampling steps T

for t = 1:T **do**

$(\beta^{(t)}, \{\pi_\ell^{(t)}\}_{\ell=1}^{\infty}) \leftarrow f\left(\beta, \{\pi_\ell\}_{\ell=1}^{\infty} \mid \{C_i^{(t-1)}\}_{i=1}^{n}\right)$ via double MH algorithm

$u_i^{(t)} \leftarrow \text{Unif}(0, \pi^{(t)}_{C_i^{(t-1)}})$ for all $i = 1, \cdots, n$

$C_i^{(t)} \leftarrow \mathbb{P}(C_i \mid u_i^{(t)}, \{\pi_\ell^{(t)}\}_{\ell=1}^{\infty}, \{\eta_{h\ell}^{(t-1)}\}_{h=1}^{H}, \{\sigma_h^{2(t-1)}\}_{h=1}^{H})$ for all $i = 1, \cdots, n$

$\eta_{h\ell}^{(t)} \leftarrow \text{N}(\widetilde{a}_{\eta,h\ell}, \widetilde{b}^2_{\eta,h\ell})$, where $(\widetilde{a}_{\eta,h\ell}, \widetilde{b}^2_{\eta,h\ell})$ are calculated based on $\{C_i^{(t)}\}_{i=1}^{n}$ and $\sigma_h^{2(t-1)}$

$\sigma_h^{2(t)} \leftarrow \text{inv-}\Gamma\,(\widetilde{\kappa}, \widetilde{\tau})$, where $(\widetilde{\kappa}, \widetilde{\tau})$ are calculated based on $\{C_i^{(t)}\}_{i=1}^{n}$ and $\eta_{h\ell}^{(t)}$

end for

Output:

Posterior samples of the parameters

Use the posterior mode of $\{C_i^{[(b+1):T]}\}$ as the estimated cluster label of cell i $(1 \le i \le n)$

S2 Implementation details of all the methods

- BACT: For BACT, we set the initial cell cluster number as the number of underlying ground truth provided by Yuan et al. (2024) for the STARmap* and MERFISH datasets, and as the number of cell layers provided by Singhal et al. (2024) for the Slide-seq dataset, aiming to prevent overfitting that may arise from a large initial cluster number and to facilitate a more effective estimation of the heterogeneous gene expression profiles across different cell types. We note that the initial cell cluster number only serves as initialization and is not necessarily equal to the final estimated cluster number. The number of neighbors is set to six and the number of PCs is set to 50 in all the real applications. The remaining parameters are set to their default values.

  The arguments in the main function "BACT" include preprocessed gene expression principal component matrix (gene_data_pc), spatial coordinates of cells (coord), spatial sequencing platform (platform), initial cell type number (num_init), number of neighbors for each cell (num_nei), mean of the normal prior for $\eta_{h\ell}$ (a_eta), standard deviation of the normal prior for $\eta_{h\ell}$ (b_eta), shape parameter of the inverse gamma prior for σ_h^2 (IGkappa), scale parameter of the inverse gamma prior for σ_h^2 (IGtau), hyperparameter of the GEM distribution for the stick-breaking prior of π_ℓ (dpAlpha), mean of the normal distribution before truncation for the spatial interaction parameter β (a_beta), standard deviation of the normal distribution before truncation for β (tau_beta), standard deviation of the normal distribution before truncation for the proposal distribution of ξ_ℓ^* (tau0), standard deviation of the normal distribution before truncation for the proposal distribution of β (tau1), a relatively large fixed positive integer used to determine proposal distribution form of ξ_ℓ^* (M0), number of MCMC iterations (numOfMCMC), number of iterations as burn-in (burnIn), a boolean value that determines whether β is fixed at zero (Is_beta_zero), a boolean value that determines whether warm start steps by KMeans are used to initialize C (Is_warm_start), a boolean value that determines whether the results by KMeans are used to initialize mean and standard deviation of each cluster (Is_kmeans_use_mean_sd), a boolean value that determines whether iteration information during model training are printed (Is_print), length of iteration interval to print the number of iterations (print_gap), a boolean value that determines whether a random seed is used for reproducibility (Is_random_seed), and a random seed (random_seed).

- SpaGCN: SpaGCN is a cell typing algorithm based on graph convolutional network (Hu et al., 2021), and its Python implementation code is publicly accessible via GitHub `https://github.com/jianhuupenn/SpaGCN`. Since the original code includes preprocessing steps for raw count data, we made a few adjustments to enable the updated code to be directly applied to the normalized gene expression data (for example, the MERFISH dataset). We set the prespecified clustering number as the number of annotated cell types. Since the datasets used in the main text do not contain image data, we set the argument "img" to zero. The other parameters were maintained at their default settings.

  The arguments in the main function "detect_spatial_domains_ez_mode" include ST raw count matrix (adata), histology image data (img), spot and pixel coordinates (x_array, y_array, x_pixel, y_pixel), predetermined clustering number (n_clusters), weight of histology

(s, its default is one), size of the square that contains image pixels (b), number of total epochs (max_epochs), and number of selected top principle components after running the principal component analysis (num_pcs).

- STAGATE is an approach for cell typing that employs graph attention autoencoders to derive low-dimensional latent embeddings of cells (Dong and Zhang, 2022). The Python code for STAGATE can be accessed publicly on GitHub https://github.com/zhanglabtools/STAGATE. Normalization and logarithmic transformation of the raw data matrix are performed before users implement the main function "train_STAGATE". Following the guidelines provided by Dong and Zhang (2022), we did not incorporate the cell-type-aware spatial neighbor network by setting the alpha parameter to its default value of zero. The spatial network was constructed by selecting k nearest neighbors of each cell with $k = 6$. We assigned the predefined number of clusters to the annotated cell type number. All other parameters are maintained at their default settings. For cell clustering, STAGATE utilizes the "mclust_R" function based on the R package mclust.

  The arguments in the main function "train_STAGATE" include the preprocessed gene expression data with coordinate information (adata), the weight of cell type-aware spatial neighbor network (alpha, its default is zero), and number of total epochs in training (n_epochs).

- BANKSY: BANKSY performs cell typing through the cell embedding in a product space of local neighborhood transcriptomics (Singhal et al., 2024). The Python code of BANKSY is publicly available on GitHub https://github.com/prabhakarlab/Banksy_py. Initialization is conducted by the function "initialize_banksy" based on the k nearest neighbors (argument: k_geom) before implementing the model. The number of neighbors is set to six and the number of PCs is set to 50 in all the real applications. We set the prespecified clustering number as the number of annotated cell types provided by the cell type annotation information. The remaining parameters are set to their default values. We employs "mclust_R" as the clustering algorithm for BANKSY using R package mclust.

  The arguments in the main function "run_banksy_multiparam" include AnnData object of the ST data (adata), dictionary object storing the spatial graph obtained after initializing the BANKSY object (banksy_dict), lambda value (lambda_list), clustering resolution for Leiden clustering (resolutions), color list for plotting (color_list), integer determines whether using AGF matrix (max_m), the output file path (filepath), keys of coordinates (key), number of principal components (pca_dims), key of cell type annotation (annotation_key), predetermined number of clusters (max_labels), used algorithm for clustering (cluster_algorithm), a boolean value determines if labels are kept consistent across different hyperparameter settings when using Leiden clustering (match_labels), a boolean value determines if the result figure is saved (savefig), a boolean value determines if the nonspatial banksy matrix is added (add_nonspatial), and a boolean value determines if the model needs to balance the variance of the cell's expression with the variance of the neighbor expression matrix (variance_balance).

- BASS: BASS is able to perform cell type identification and domain detection simultaneously with multiple tissue sections (Li and Zhou, 2022). The R code of BASS is publicly

available on GitHub https://github.com/zhengli09/BASS. Specifically, we first create a BASS object by the function "createBASSObject" with the number of neighbors set to six (argument: k) and the numbers of cell types (argument: C) and regions (argument: R) determined according to the annotation of the data. We then preprocess the raw data by the function "BASS.preprocess" via the principal component analysis and obtain the top 50 PCs (argument: nPC). The remaining parameters are set to their default values.

The argument in the main function "BASS.run" only includes the BASS object created and preprocessed by the functions introduced above (BASS), and the arguments used in the procedure of the main function include the initialization method for cell typing and domain detection (init_method), the scale parameter of the inverse Wishart prior on the variance-covariance matrix (psi0), degrees of freedom of the inverse Wishart prior (n0), concentration parameter of the Dirichlet prior assigned on the cell type composition (alpha0), number of burn-in steps in the MCMC process (burnin), total number of posterior sampling steps in the MCMC process (nsample), ways to specify the cell-cell interaction parameter (beta_method), the prespecified or initial value of the cell-cell interaction parameter (beta), step size of the uniform random walk (step_size), number of burn-in steps in the sampling of Potts model (potts_burnin), and number of Potts samples to approximate the partition ratio (potts_nsample).

S3 Additional cell typing analysis in the mouse visual cortex STARmap* data

In the analysis of the STARmap* dataset, BACT identified 18 distinct cell types, while Wang et al. (2018) provided annotations for only 16 cell types. To further investigate the additional cell type clusters, we first generated a heatmap based on the estimated and annotated cell types, with each row representing the proportion of estimated cell clusters within each annotated cell type, which is shown in Figure S4. We observed that microglia (micro) cells primarily contain clusters C10 and C18 (with more than five cells). We then used the R package edgeR to identify differentially expressed (DE) genes in both C10 and C18, and uploaded all DE genes into the GSEA platform for pathway analysis.

For estimated cell cluster C10, we identified a statistically significant pathway "GOCC_ACTIN_BASED_CELL_PROJECTION," that is essential for the structural and functional roles of microglia (micro) cells, as these cells rely on actin-supported projections for motility, environmental sensing, and immune surveillance within the central nervous system. In contrast, the DE genes in estimated cluster C18 were enriched in 74 significant GOBP pathways, which are closely associated with the regulatory roles of micro cells in neurodevelopmental processes. These observations suggest that although cells in both C10 and C18 belong to micro cells, they may represent distinct subtypes with different biological functions. This also indicates that BACT has the capability to identify more latent cell subtypes.

S4 Different parameter settings for competing methods in the STARmap* data analysis

To illustrate the consistency of results across all competing methods, we selected three distinct parameter configurations for each method and conducted five random repeats.

- For SpaGCN, we set the parameter "p" to 0.5, 0.6, and 0.8, respectively, where "p" denotes the relative contribution of neighboring spots or cells to the total gene expression.
- To evaluate the results of STAGATE, we set the parameters "lr" and "weight_decay" to equal values of 5×10^{-5}, 1×10^{-4} and 2×10^{-4}, respectively, where "lr" and "weight_decay" represent the learning rate and the weight decay for the optimizer AdamOptimizer.
- To implement BANKSY, we assign the mixing parameter "λ" values of 0.7, 0.8, and 0.9, respectively. Here, "λ" measures the weights of the individual gene expression and expressions from the neighboring cells.
- We specify the parameters "(psi0, n0, alpha0, step_size)" in BASS model as (1,1,1,0.1), (1.5,1.5,1.5,0.01), and (2,2,2,0.05), respectively. "psi0" and "n0" are hyperparameters involved in the inverse Wishart prior on the variance-covariance matrix, denoting the scale and degree of freedom, respectively, "alpha0" is the concentration parameter of the Dirichlet prior on the cell type composition, and "step_size" determines the step size of a uniform random walk.

Figure S3 shows the ARI boxplots derived from these random experiments for the competing methods, demonstrating that their ARI values under different parameter settings are similar. Thus, the cell typing performances of these methods are consistent for STARmap* data, and are relatively less accurate than that of BACT with an ARI value of 0.629 recorded in Figure 2(c) in the main text.

S5 Implementation of the competing methods with the prespecified cell type number being 18

To verify the facilitation in cell type identification by using the cell type number detected by BACT, we set the prespecified cell type number to 18 for other competing methods, as BACT identified 18 distinct cell types in this real application, and conducted five random repeats for all the methods. The ARI boxplots are displayed in Figure S5. When the predetermined cell type number was 16, the ARI median values of SpaGCN, STAGATE, BANKSY and BASS are 0.436, 0.301, 0.113 and 0.304, respectively. When the cell type number was changed to 18, the ARI median values of these methods increased to 0.455, 0.306, 0.119, 0.326, respectively. Therefore, using the cell type number learned by BACT as the predetermined cell type number can improve the cell typing performance of other methods.

S6 Performance of BACT for Slide-seq data with a lower clustering resolution

The spatial interaction parameter in BACT captures the strength of spatial dependency between cells, with a larger value indicating that neighboring cells are more likely to share the same cell type indicators, thus resulting in fewer cell clusters. In our model, we assigned the spatial interaction parameter a truncated normal prior, which represents a normal distribution $\mathrm{N}(a_\beta, \tau_\beta^2)$ truncated on the the domain of positive real numbers. In the application to Slide-seq dataset, we originally set a_β to be one in the main text, and changed it to be three in this section to investigate the cell typing performance with stronger prior spatial interactions. Figure S6 demonstrates the five repeating results of BACT, showing that it indeed detected fewer cell clusters but still successfully identified the choroid plexus structure in all repeats. On the other hand, due to the lower clustering resolution, the annotated cell types granule cell layer and molecular layer were merged into a single type in repeat one, and granule cell layer and molecular layer were merged into a single type in repeat two to five. Notice that these cell layers were successfully recovered by BACT with higher resolution in Figure 4(b) in the main text.

In terms of biological functions, the purkinje layer, granule cell layer and molecular layer are more similar in their cell type compositions, as they are components of the cerebellar cortex and collaborate closely together in cerebellar motor control and information processing. In contrast, the choroid plexus, located in the ventricles, primarily consists of choroid plexus epithelial cells, showing structural and cellular differences from the cerebellar cortex. Therefore, compared to the cell typing results of STAGATE, BACT identifies the heterogeneous characteristics of the choroid plexus at both high and low clustering resolutions more effectively, and maintains better spatial coherence than SpaGCN. BANKSY and BASS, however, could only partially identify certain cell layers.

S7 Human dorsolateral prefrontal cortex data analysis

BACT is specifically designed for single-cell resolved ST data, but in a statistical sense, it can also be applied to spot-resolution ST data, such as the human dorsolateral prefrontal cortex (DLPFC) data collected by Maynard et al. (2021), and is available at the 10x Genomics Visium platform. The dataset consists of 12 manually annotated tissue sections, and we selected DLPFC section 151507 for analysis and visualization. The domain detection performances of all methods are quantitatively compared based on the domain structure annotation (Figure S7(a)). Figures S7(b)-(f) illustrate the domain detection results of BACT, SpaGCN, STAGATE, BANKSY, and BASS, respectively. BACT effectively identified the main parts of Layers 1 and 3, but failed to clearly capture other regions, resulting in an ARI of 0.429. This value is comparable to the ARI value of SpaGCN (0.427), where SpaGCN performed well in capturing the domain structure of Layers 1, 2, and 3, but the estimated regions lacked spatial continuity. Among all methods, STAGATE achieved the highest ARI (0.520), excelling in both region identification accuracy and spatial continuity. In contrast, BANKSY and BASS obtained relatively lower ARI values. Specifically, BANKSY (ARI=0.399) incorrectly clustered main

parts of spots belonging to Layers 3, 4, and 5 into estimated spot cluster C5, and spots belonging to Layer 6 and WM into C6. Similarly, BASS (ARI=0.409) inaccurately merged parts of Layers 3, 4, and 5 into C3 and parts of Layers 5, 6, and WM into C5. The ARI boxplots for all the methods based on the five random repeats are demonstrated in Figure S8.

As the primary task for the spot-resolution ST data is to uncover domain structures rather than identifying cell types, it is expected that BACT may not perform as well as methods tailored for domain detection. Moreover, the domain detection task has been studied in our previous work of BINRES (Yan and Luo, 2024), which was specifically developed to carry out region segmentation for spot-level ST data, and we conducted a more comprehensive investigation of spot-resolution ST data in this work.

Significant pathways
GOBP_NEGATIVE_REGULATION_OF_CATION_CHACHANNEL_ACTIVITY
GOBP_POSITIVE_REGULATION_OF_CALCIUM_IO_ION_TRANSMEMBRANE _TRANSPORTER_ACTIVITY
GOBP_POTASSIUM_ION_IMPORT_ACROSS_PLASMASMA_MEMBRANE
GOBP_RESPONSE_TO_VITAMIN
GOCC_APICAL_PART_OF_CELL
GOCC_APICAL_PLASMA_MEMBRANE
GOCC_CATION_CHANNEL_COMPLEX
**GOCC_EXTRACELLULAR_SPACE**
GOCC_MONOATOMIC_ION_CHANNEL_COMPLEX
GOCC_PLASMA_MEMBRANE_REGION
GOCC_POTASSIUM_CHANNEL_COMPLEX
GOCC_SECRETORY_VESICLE
GOCC_TRANSPORTER_COMPLEX
GOCC_VOLTAGE_GATED_POTASSIUM_CHANNEL_CL_COMPLEX
GOMF_GATED_CHANNEL_ACTIVITY
GOMF_VOLTAGE_GATED_POTASSIUM_CHANNEL_AL_ACTIVITY
GOMF_POTASSIUM_CHANNEL_ACTIVITY
GOMF_LIGAND_GATED_CHANNEL_ACTIVITY
GOMF_VOLTAGE_GATED_MONOATOMIC_CATION_CN_CHANNEL_ACTIVITY
GOBP_EXPORT_FROM_CELL
GOMF_POTASSIUM_ION_TRANSMEMBRANE_TRANSANSPORTER_ACTIVITY
GOMF_PASSIVE_TRANSMEMBRANE_TRANSPORTERTER_ACTIVITY
GOMF_TRANSMEMBRANE_TRANSPORTER_BINDING
GOMF_INWARD_RECTIFIER_POTASSIUM_CHANNENNEL_ACTIVITY
GOMF_VOLTAGE_GATED_CHANNEL_ACTIVITY
GOMF_SALT_TRANSMEMBRANE_TRANSPORTER_AC_ACTIVITY
GOMF_INORGANIC_MOLECULAR_ENTITY_TRANSMNSMEMBRANE _TRANSPORTER_ACTIVITY
GOMF_ORGANIC_ACID_BINDING
GOMF_ISOPRENOID_BINDING
GOMF_MONOATOMIC_ION_TRANSMEMBRANE_TRANRANSPORTER _ACTIVITY

Table S1: Significant pathways obtained from the platform GSEA based on the 15 identified DE genes. The bold pathway name corresponds to the mouse brain structure choroid plexus shown in Figure 4(a) in the main text.

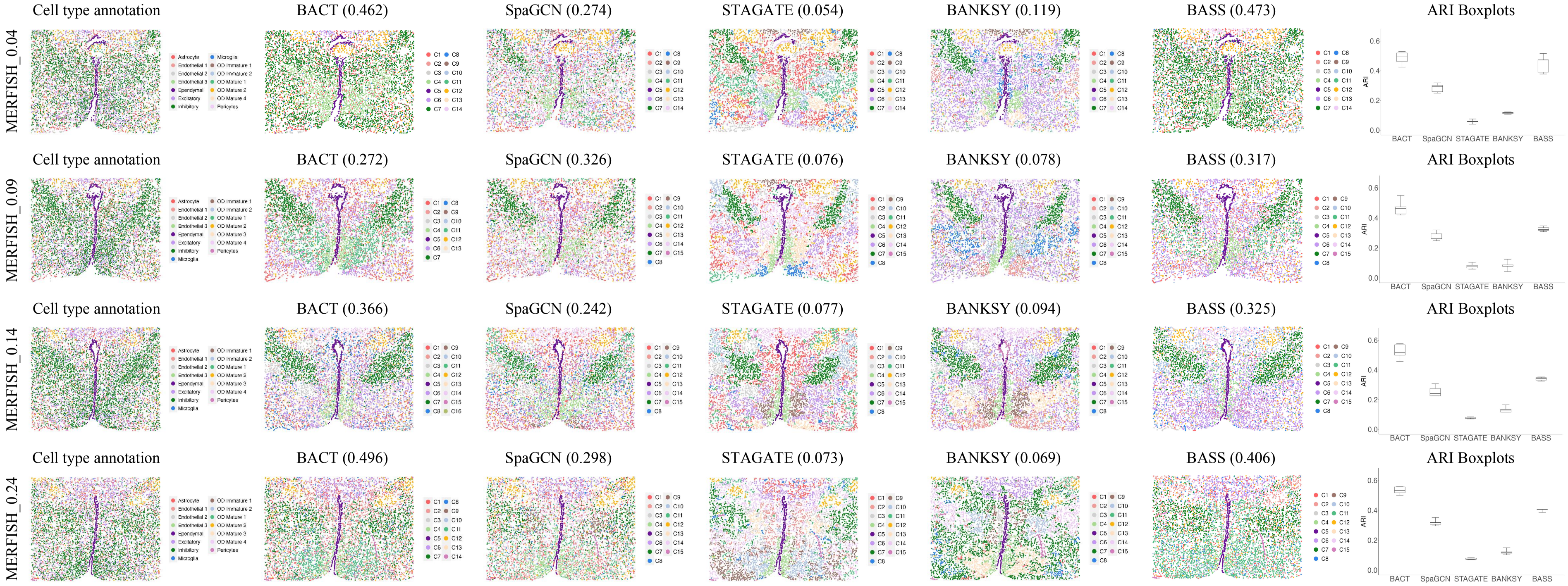

Figure S1: Underlying cell type annotations, the cell typing performances of BACT and competing methods, and the ARI boxplots of all methods based on five random repeats for the mouse hypothalamic preoptic region datasets MERFISH_0.04, MERFISH_0.09, MERFISH_0.14, and MERFISH_0.24

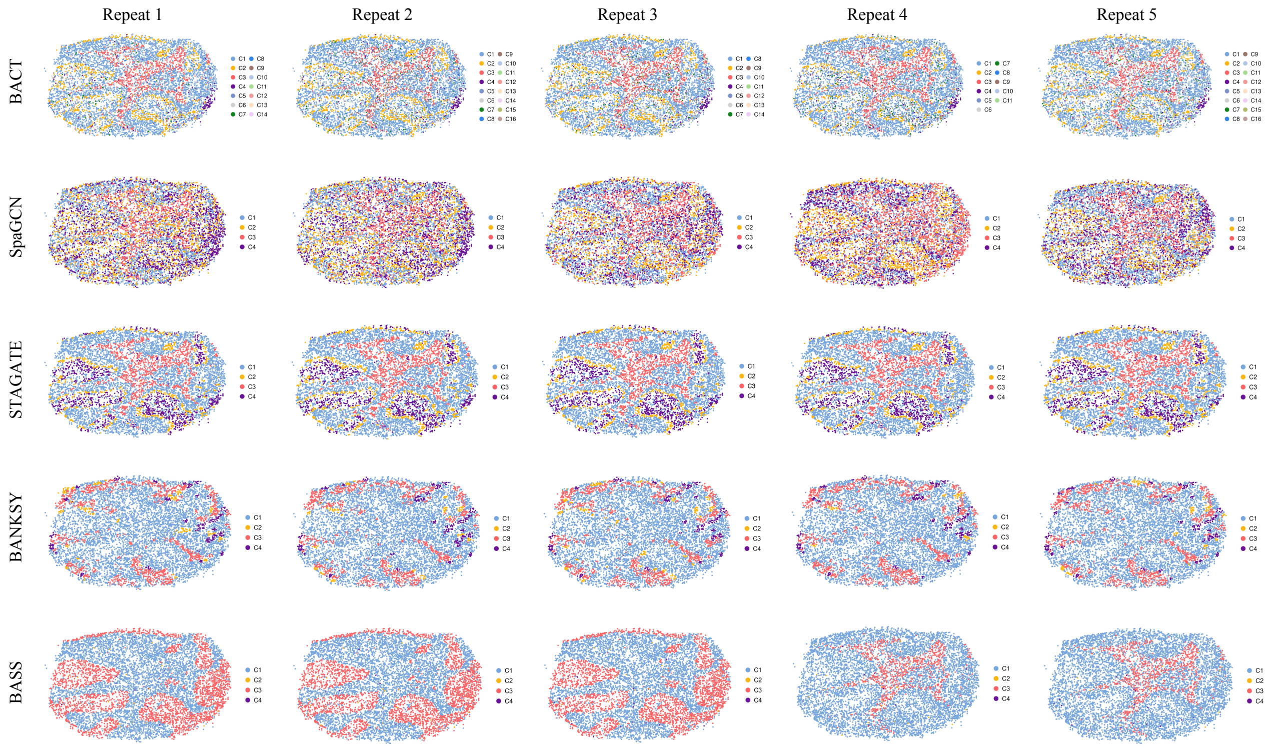

Figure S2: The cell typing results of BACT and competing methods based on five random repeats for the mouse cerebellum Slide-seq data.

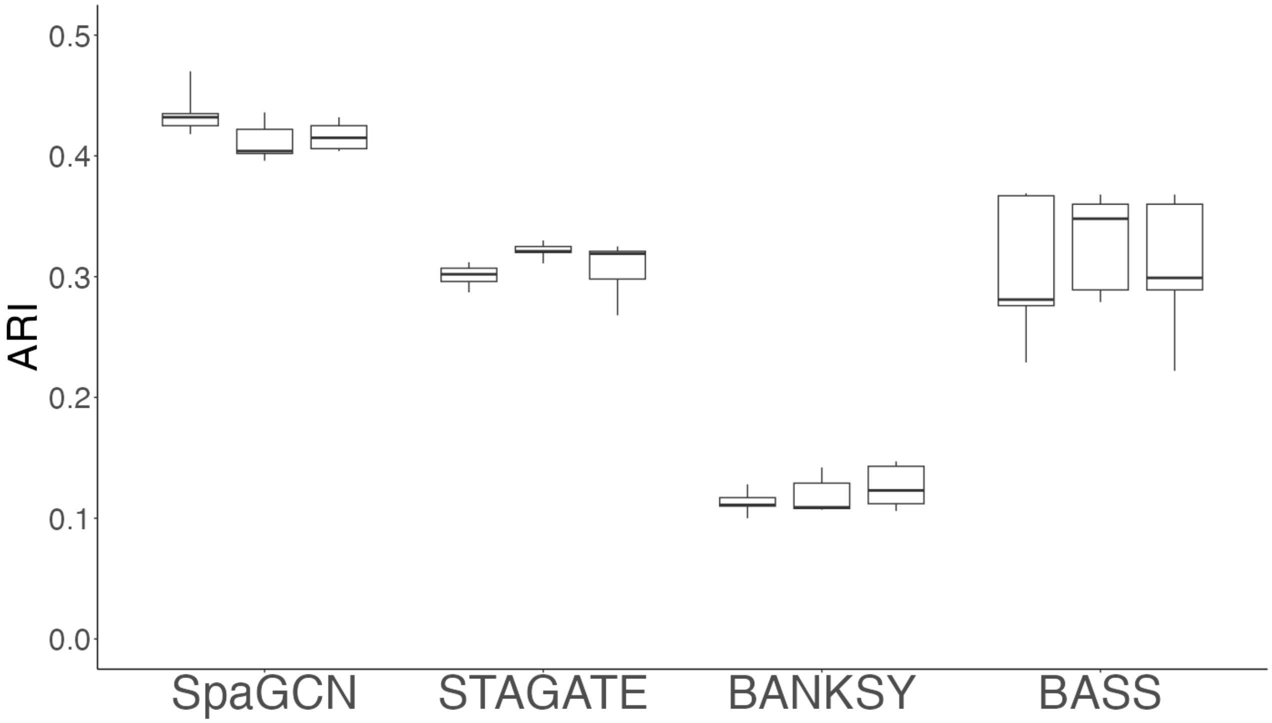

Figure S3: The ARI boxplots for all the competing methods based on different settings of parameters.

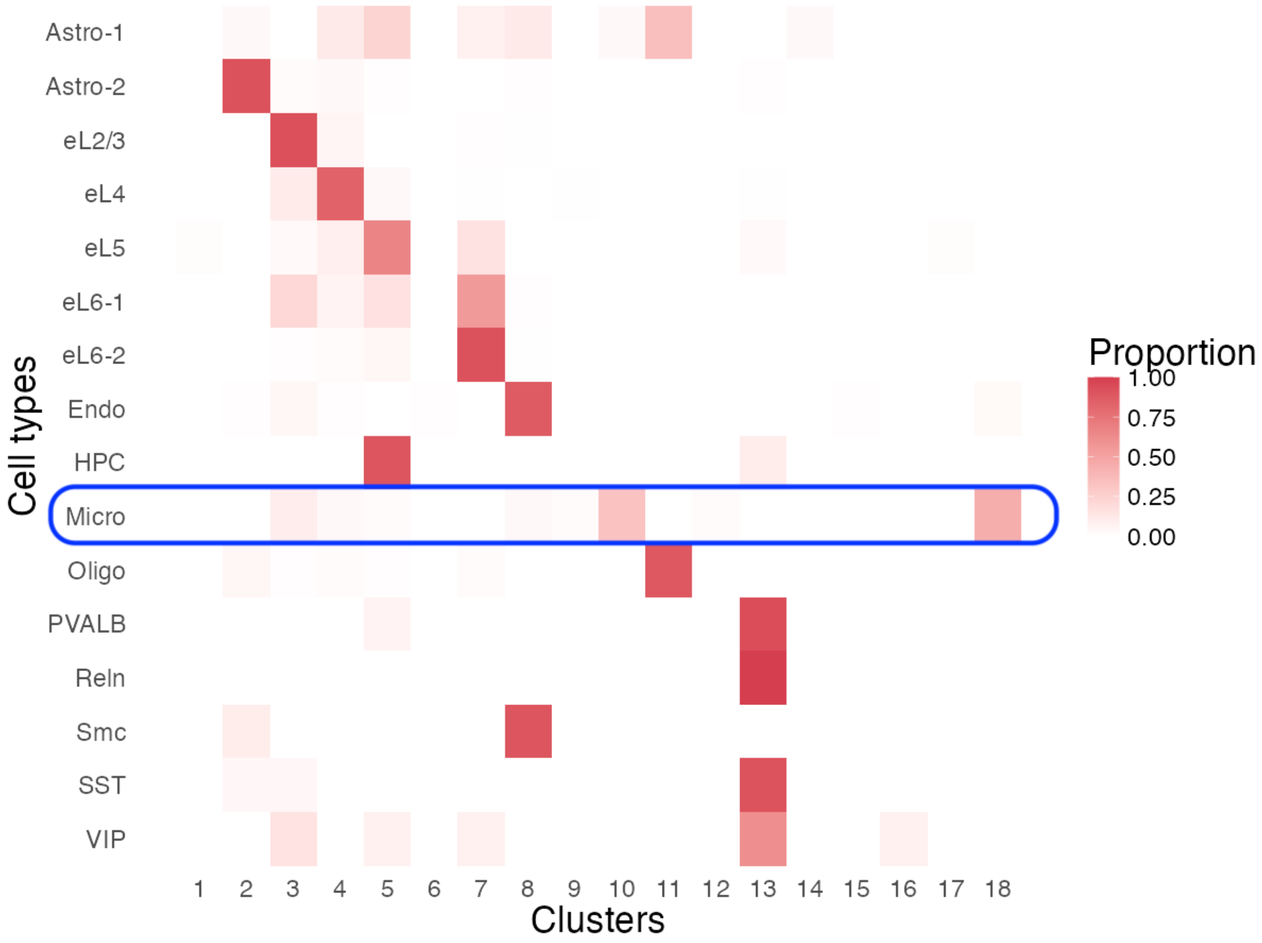

Figure S4: The heatmap for the proportions of estimated cell clusters identified by BACT in the analysis of the mouse visual cortex STARmap* data. Each row represents the estimated cell cluster proportions within each annotated cell type.

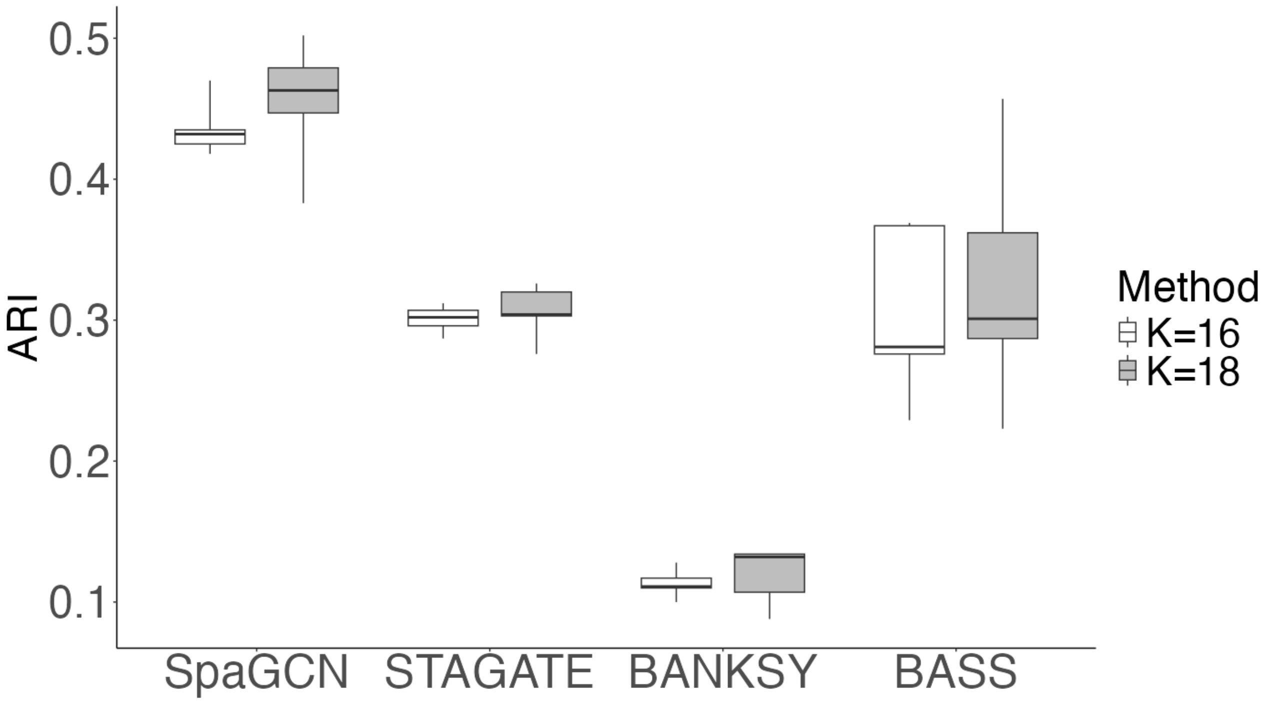

Figure S5: The ARI boxplots for all the competing methods based on the predetermined cell type number being 16 and 18, respectively. K denotes the specific cell type number used for these methods.

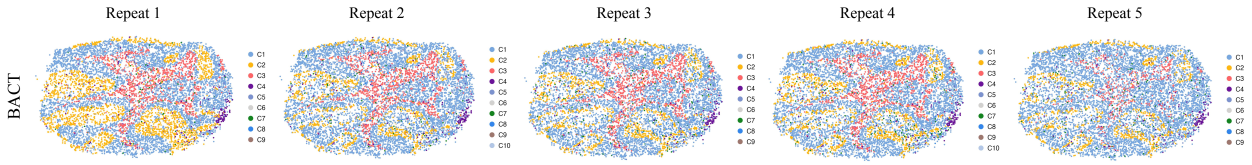

Figure S6: The cell typing performances of BACT based on five random repeats.

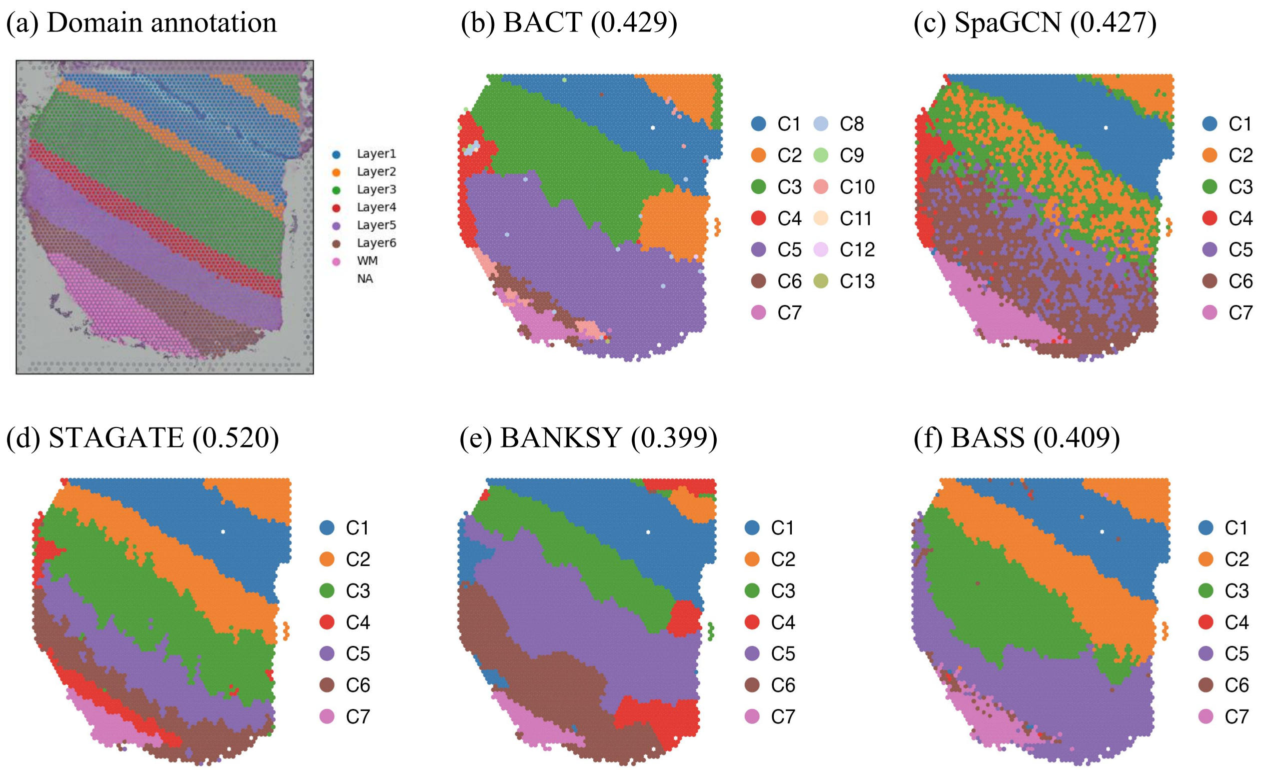

Figure S7: (a) Manual domain annotation for the human dorsolateral prefrontal cortex section 151507 data. The cell typing performances are shown for (b) BACT, (c) SpaGCN, (d) STAGATE, (e) BANKSY, and (f) BASS, where the number in the parentheses is the corresponding ARI value which applies to other panels.

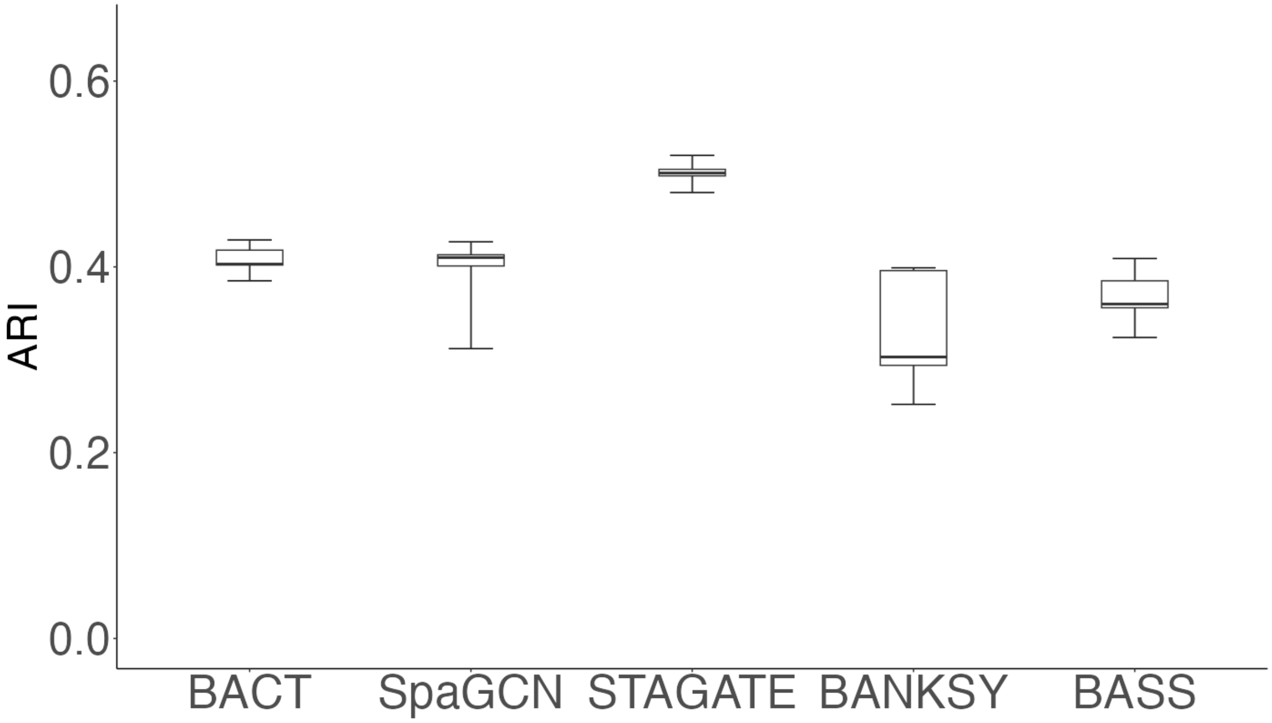

Figure S8: The ARI boxplots of all methods based on five random repeats for the human dorsolateral prefrontal cortex section 151507 data.

References

Dong, K. and S. Zhang (2022). Deciphering spatial domains from spatially resolved transcriptomics with an adaptive graph attention auto-encoder. *Nature Communications 13*(1), 1–12.

Hu, J., X. Li, K. Coleman, A. Schroeder, N. Ma, D. J. Irwin, E. B. Lee, R. T. Shinohara, and M. Li (2021). SpaGCN: Integrating gene expression, spatial location and histology to identify spatial domains and spatially variable genes by graph convolutional network. *Nature Methods 18*(11), 1342–1351.

Li, Z. and X. Zhou (2022). BASS: multi-scale and multi-sample analysis enables accurate cell type clustering and spatial domain detection in spatial transcriptomic studies. *Genome Biology 23*(1), 168.

Maynard, K. R., L. Collado-Torres, L. M. Weber, C. Uytingco, B. K. Barry, S. R. Williams, J. L. Catallini, M. N. Tran, Z. Besich, M. Tippani, J. Chew, Y. Yin, J. E. Kleinman, T. M. Hyde, N. Rao, S. C. Hicks, M. Keri, and J. A. E. (2021). Transcriptome-scale spatial gene expression in the human dorsolateral prefrontal cortex. *Nature Neuroscience 24*(3), 425–436.

Singhal, V., N. Chou, J. Lee, Y. Yue, J. Liu, W. K. Chock, L. Lin, Y.-C. Chang, E. M. L. Teo, J. Aow, et al. (2024). BANKSY unifies cell typing and tissue domain segmentation for scalable spatial omics data analysis. *Nature Genetics 56*(3), 431–441.

Van Dyk, D. A. and T. Park (2008). Partially collapsed Gibbs samplers: Theory and methods. *Journal of the American Statistical Association 103*(482), 790–796.

Wang, X., W. E. Allen, M. A. Wright, E. L. Sylwestrak, N. Samusik, S. Vesuna, K. Evans, C. Liu, C. Ramakrishnan, J. Liu, G. P. Nolan, F.-A. Bava, and K. Deisseroth (2018). Three-dimensional intact-tissue sequencing of single-cell transcriptional states. *Science 361*(6400), eaat5691.

Yan, Y. and X. Luo (2024). Bayesian integrative region segmentation in spatially resolved transcriptomic studies. *Journal of the American Statistical Association 119*(547), 1709–1721.

Yuan, Z., F. Zhao, S. Lin, Y. Zhao, J. Yao, Y. Cui, X.-Y. Zhang, and Y. Zhao (2024). Benchmarking spatial clustering methods with spatially resolved transcriptomics data. *Nature Methods 21*(4), 712–722.
